# Recent Development of Endo‐hepatology

**DOI:** 10.1002/deo2.70404

**Published:** 2026-08-13

**Authors:** Akira Yamamiya, Atsushi Irisawa, Yasunori Inaba, Rafael Romero‐Castro

**Affiliations:** ^1^ Department of Gastroenterology Dokkyo Medical University School of Medicine Mibu Japan; ^2^ Division of Gastroenterology Virgen Macarena University Hospital Seville Spain

**Keywords:** endo‐hepatology, endoscopic ultrasound, EUS‐guided portal pressure gradient measurement, interventional EUS, portal hypertension

## Abstract

Endo‐hepatology is a rapidly evolving field encompassing endoscopic diagnosis and treatment of hepatobiliary diseases, primarily using endoscopic ultrasound (EUS). Since its conceptualization in 2012, the field has expanded dramatically alongside advances in interventional EUS. A landmark 2024 review broadened the concept to include endoscopic management of hepatobiliary diseases using interventional EUS/endoscopic retrograde cholangiopancreatography and advanced imaging technologies. In portal hypertension (PH), diagnostic applications include EUS‐based variceal hemodynamic assessment, EUS‐guided shear wave elastography, EUS‐guided porto‐systemic pressure gradient measurement (EUS‐PPGM), EUS‐guided liver biopsy (EUS‐LB), and portal vein sampling. Therapeutic applications include EUS‐guided vascular embolization for gastric, esophageal, and ectopic varices; portosystemic shunt obliteration for refractory hepatic encephalopathy; radiofrequency ablation of hepatocellular carcinoma; and emerging procedures such as intrahepatic portosystemic shunt creation and portal vein stenting. EUS‐PPGM enables direct, minimally invasive portal pressure measurement and correlates strongly with the hepatic venous pressure gradient (HVPG). It may offer particular advantages in pre‐sinusoidal PH, such as porto‐sinusoidal vascular disorder, and in conditions with mixed sinusoidal and presinusoidal components, including metabolic dysfunction‐associated steatotic liver disease, where HVPG may underestimate portal pressure. The “one‐stop” concept integrates portal pressure measurement, LB, liver stiffness assessment, and variceal evaluation and treatment within a single endoscopic session. EUS‐guided coil plus cyanoacrylate injection has demonstrated favorable efficacy and safety for gastric varices, while EUS‐LB provides diagnostic accuracy comparable to percutaneous biopsy with less post‐procedure pain. By integrating diagnostic and therapeutic procedures, endo‐hepatology bridges endoscopy and hepatology and may provide comprehensive, minimally invasive, and personalized management of PH and chronic liver disease.

## 1 Introduction

The term “Endo‐hepatology” was coined by Professor Kenneth J. Chang in 2012, based on the concept that the integrated management of portal hypertension (PH) and liver diseases by hepatologists and endoscopists working collaboratively is ideal [1]. Initially, the term referred specifically to endoscopic care of liver diseases using endoscopic ultrasound (EUS). A comprehensive 2024 review [2] further broadened the definition to encompass all endoscopic management of hepatobiliary diseases utilizing interventional EUS/endoscopic retrograde cholangiopancreatography and advanced imaging technologies, including EUS‐based liver stiffness measurement, hemodynamic assessment of varices, portal pressure measurement, LB, variceal therapy, tumor ablation, biliary drainage, and cholangioscopy for primary sclerosing cholangitis or post‐transplant biliary complications.

Portal hypertension is a common and serious complication of advanced chronic liver disease (CLD), being the main driver of life‐threatening complications. Accurate hemodynamic assessment is central to risk stratification, treatment planning, and prognostication in patients with CLD. The gold‐standard method to estimate the portal vein pressure is the hepatic venous pressure gradient (HVPG), a surrogate of the wedged hepatic venous pressure due to the impossibility of accessing the portal vein. However, HVPG is invasive, technically demanding, and unable to accurately reflect portal pressure in pre‐sinusoidal forms of PH. Therefore, it is not recommended in daily clinical practice, depriving the majority of patients of their potential benefits. Endo‐hepatology offers a unique platform bringing endoscopic precision to bear on hepatological problems previously managed exclusively by hepatologists and interventional radiologists, offering the prospect of comprehensive assessment and treatment within a single endoscopic session. In this review, we focus on EUS‐based endo‐hepatology in the context of PH management and provide a comprehensive overview of its current status and future perspectives.

## 2 Diagnostic Endo‐Hepatology in PH

### 2.1 Assessment of Esophagogastric Variceal Hemodynamics

Before the term “endo‐hepatology” was formally introduced, EUS and ultrasound mini‐probes (UMP) had already demonstrated value for minimally invasive characterization of portal hemodynamics and variceal anatomy through the gastrointestinal wall. Since the pioneering observations of Caletti et al., who first reported EUS detection of PH and esophageal varices (EV) in 1986, numerous studies have characterized variceal hemodynamics using EUS‐based modalities [3, 4].

**FIGURE 1:**
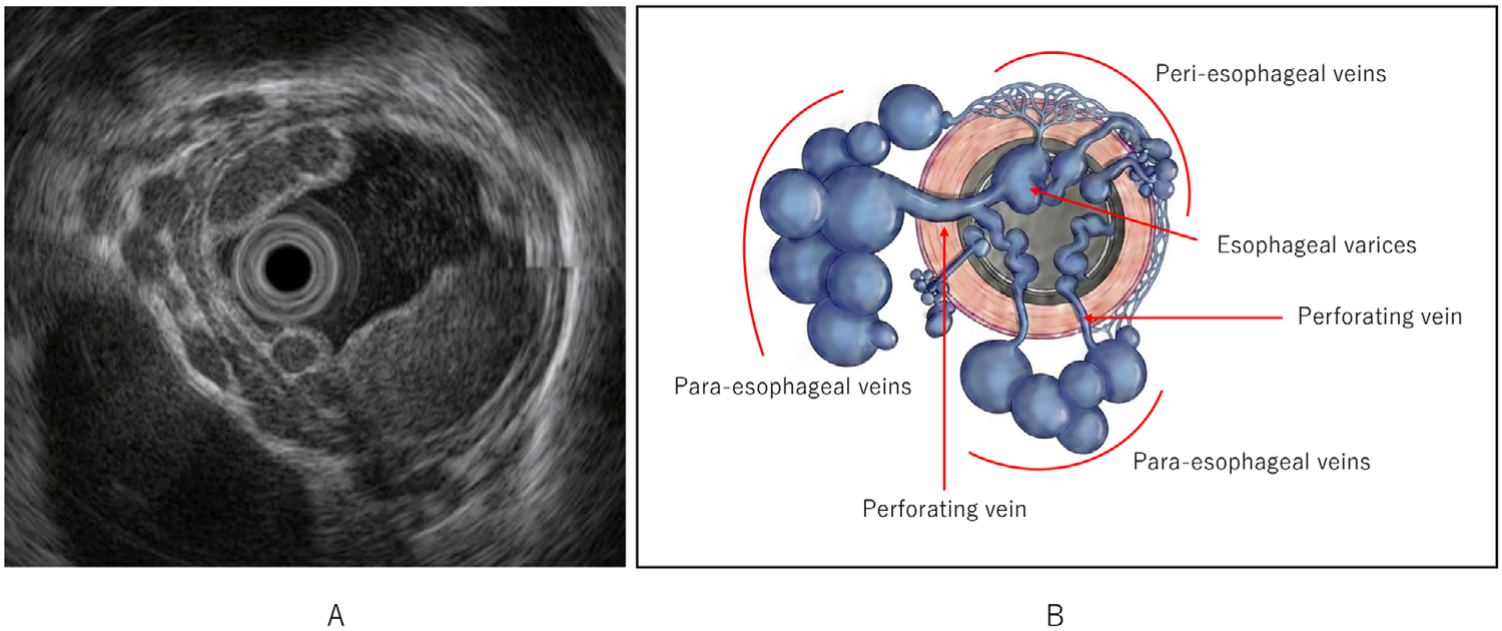
EUS image of esophageal varices and illustrated schema A: Ultrasound mini‐probe revealed hemodynamics with various vessels around the esophageal wall in detail. B: The scheme of EUS analysis of esophageal varices around the esophageal wall in patients with portal hypertension.

Irisawa et al. analyzed the local hemodynamics of EV using UMP, identifying three collateral vessel types: perforating veins (Pv), peri‐esophageal veins (Peri‐v), and para‐esophageal veins, and demonstrating that Pv and Peri‐v are significantly associated with endoscopic recurrence after endoscopic injection of sclerosants (EIS) in EV [5, 6]. This work highlighted the concept that variceal hemodynamics, not just variceal morphology, should guide treatment planning. Sato et al. subsequently demonstrated with color Doppler EUS that Pv in the lower esophagus serve primarily as afferent vessels feeding EV, contributing to their development and maintenance [7]. Nakamura et al. classified local EV hemodynamics into four morphological patterns using three‐dimensional UMP and proposed EUS‐based indications for EIS and endoscopic variceal ligation (EVL) according to the predominant hemodynamic pattern [8]. These contributions represent the historical foundation of EUS‐based endo‐hepatology in Japan, establishing the principle that real‐time vascular imaging can and should guide therapeutic decision‐making in PH (Figure 1).

### 2.2 Liver Stiffness Measurement: EUS‐guided Shear Wave Elastography

**FIGURE 2:**
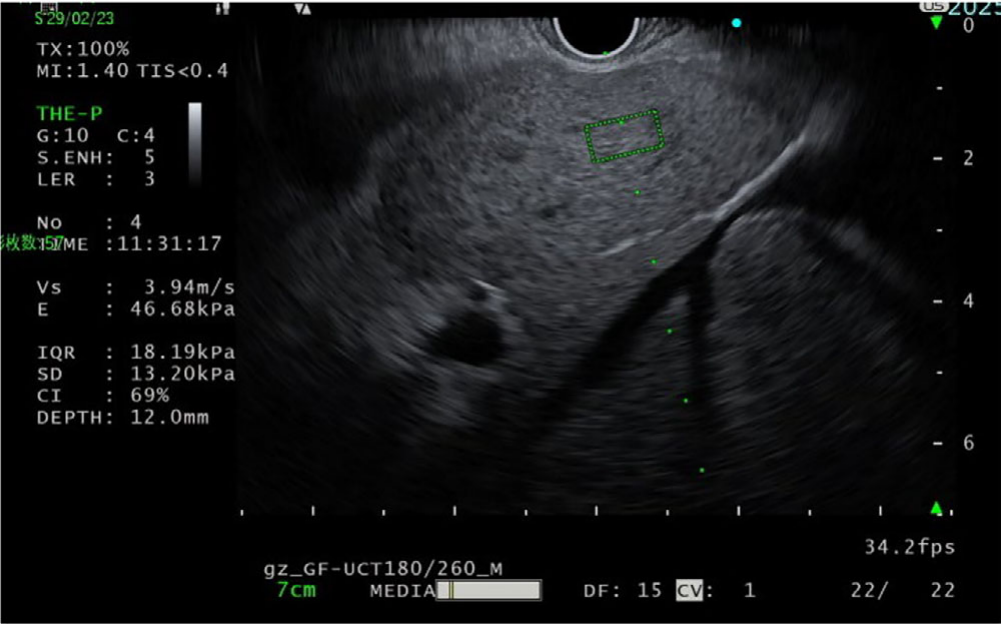
The figure showed EUS‐SWE (EU‐ME3, Olympus) measurements, with 10 measurements taken and the average calculated.

Shear wave elastography (SWE), measuring the propagation velocity of transverse shear waves through liver parenchyma as a surrogate for tissue stiffness, is now a widely validated non‐invasive fibrosis assessment tool. Transcutaneous vibration‐controlled transient elastography (VCTE; FibroScan) is technically limited by obesity, ascites, narrow intercostal spaces, or inability to adequately position the probe. EUS‐SWE leverages the proximity of the echoendoscope to the liver through the gastric wall, overcoming these constraints. The ability to perform SWE in the same session as other EUS‐based hepatological assessments further adds to its clinical appeal (Figure 2).

Kohli et al. compared VCTE and EUS‐SWE against LB in a pilot cohort; area under the receiver operating characteristic curve (AUROC) for cirrhosis detection was 0.90 for VCTE, 0.96 for EUS‐SWE of the left lobe, and 0.90 for the right lobe, demonstrating equivalent diagnostic accuracy [9]. Diehl et al. reported a strong correlation between right lobe EUS‐SWE and fibrosis stage (ρ = 0.571, *p* < 0.0001) in a prospective cohort, with EUS‐SWE and VCTE performing equivalently across all fibrosis stages in ROC analysis [10]. AbiMansour et al. demonstrated significant correlation between left lobe EUS‐SWE and magnetic resonance imaging elastography (MRE) values, establishing EUS‐SWE as comparable to advanced cross‐sectional imaging [11]. These data collectively support EUS‐SWE as a safe, reliable, and reproducible fibrosis assessment tool with particular value in patients unsuitable for transcutaneous elastography.

### 2.3 Portal Pressure Measurement: EUS‐guided Porto‐Systemic Pressure Gradient Measurement

Portal pressure is most commonly estimated by HVPG, calculated from the difference between wedged and free hepatic venous pressures via balloon catheter, as recommended in Baveno VII [12]. Established HVPG thresholds carry well‐defined clinical implications [13, 14, 15] (Table 1), and HVPG is a key trial endpoint: reductions achieved with non‐selective beta‐blockers (NSBB) or transjugular intrahepatic portosystemic shunt (TIPS) correlate with improved outcomes, and the acute hemodynamic response to intravenous propranolol predicts long‐term outcome, with non‐responders showing significantly higher rates of liver‐related events and mortality [16, 17]. However, HVPG requires invasive catheterization with high radiation exposure, and it reflects post‐sinusoidal resistance, making it unreliable in presinusoidal conditions such as porto‐sinusoidal vascular disorder (PSVD) and the prevalent metabolic dysfunction‐associated steatotic liver disease (MASLD), limiting its utility and motivating the search for direct alternatives.

**TABLE 1:** Prognostic significance of HVPG values.

HVPG (mmHg)	Clinical Consequences
≥6 mmHg	Portal hypertension
>10 mmHg	Clinically significant portal hypertension: increasing risk of varices rupture, refractory ascites, hepatic encephalopathy.
≥12 mmHg	Active variceal hemorrhage
≥16 mmHg	Increasing risk for liver‐related hepatic events and mortality

Non‐invasive tests (NITs) are recommended by current guidelines to detect and stratify clinically significant portal hypertension (CSPH) risk [12], but a wide gray zone remains where prevalent conditions such as MASLD, obesity, or mixed etiologies are frequently misclassified. The landmark PREDESCI study demonstrated that NSBB prevented decompensation in NIT‐identified CSPH patients with viral hepatitis or alcohol‐related CLD [18]; however, almost half of the NIT‐classified CSPH patients had an HVPG below 10 mmHg and were excluded, and the authors themselves noted that adding endoscopy or spleen stiffness measurement improved classification accuracy [19]. This likely reflects the reduced accuracy of NITs in MASLD and mixed etiologies, which cannot reliably discriminate fibrosis from inflammatory activity, underscoring the need for more accurate diagnostic tools as MASLD becomes the leading cause of CLD worldwide [20, 21].

**FIGURE 3:**
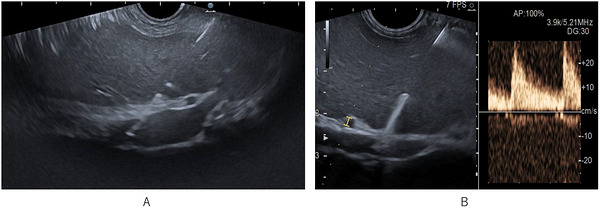
EUS‐PPG procedure. Under EUS guidance, the portal vein (A) and hepatic vein (B) were directly punctured, and portal hypertension was assessed by measuring the pressure difference. This figure used a 25G puncture needle.

EUS‐guided porto‐systemic pressure gradient measurement (EUS‐PPGM), first reported by Lai et al. in a 2004 animal model, showed high correlation (*r* = 0.91) with percutaneous catheterization [22]; subsequent refinements included intrahepatic portal vein puncture to reduce bleeding risk [23] and a digital pressure wire enabling real‐time wireless display [24]. Building on these foundations, Huang et al. developed a technique sequentially measuring portal and hepatic vein (or IVC) pressure to calculate the porto‐systemic pressure gradient (PPG) (Figure 3), with near‐perfect correlation to HVPG in animal studies (*r* = 0.985–0.99) [25] and, in a human pilot study of 28 patients, PPG values of 1.5–19 mmHg (mean 8.2 mmHg) with PPG ≥5 mmHg predicting an 18.5‐fold higher probability of cirrhosis, though hepatic vein access was difficult in 32% of patients [26]. Subsequent studies confirmed a strong correlation with HVPG (*r* = 0.923, ICC 0.82) and technical success rates of 90% or higher [27, 28].

A 2024 meta‐analysis (eight studies, 178 patients) reported pooled technical and clinical success rates of 94.6% and 85.4%, respectively, with an adverse event rate of 10.9%, mostly minor bleeding, with no procedure‐related deaths [29]. Luo et al. established clinically useful EUS‐PPGM cutoffs in 197 patients: 11.5 mmHg for variceal presence, 12.75 mmHg for decompensated cirrhosis, 15.75 mmHg for ascites, and 16.75 mmHg for variceal bleeding, correlating well with HVPG‐based benchmarks [30].

A particularly compelling application was reported by Santopaolo et al. in 26 patients with PSVD and CSPH, where mean EUS‐PPG (16.7 ± 5.5 mmHg) far exceeded mean HVPG (5.5 ± 2.8 mmHg), and EUS‐PPG, not HVPG, independently predicted hepatic decompensation [31], establishing EUS‐PPGM as a potentially transformative tool in non‐cirrhotic PH. Similarly, in morbidly obese patients undergoing combined EUS‐PPGM and EUS‐LB, Romero‐Castro et al. found EUS‐PPGM exceeded 6 mmHg in 28% of cases with neither endoscopic PH signs nor significant fibrosis, highlighting its value in detecting early, reversible liver disease [32]. The same group also demonstrated safe assessment of the acute hemodynamic response to intravenous propranolol using EUS‐PPG [33], with a prospective study of its long‐term prognostic value versus NITs currently underway.

Clinical adoption of EUS‐PPGM, currently limited in Japan, is eagerly awaited, and prospective multicenter trials comparing EUS‐PPGM with HVPG across diverse etiologies are urgently needed (Table 2). Two technical approaches exist: a dedicated 25G needle linked to a digital manometer, or a 22G needle attached to a standard invasive pressure monitoring system [27, 28]; 25G needles offer greater flexibility in narrow hepatic veins and probably lower bleeding risk [34], while a prospective comparison of both systems found the custom‐built 22G system had better pressure‐tracing quality at lower cost [35]. An international Delphi consensus has recently proposed best practices and future research directions [36]. Notably, a multicenter retrospective study of 173 patients found that portal vein pressure alone, without requiring the technically challenging hepatic vein measurement, predicted hepatic decompensation above 25 mmHg [37], a simplification that awaits prospective validation.

**TABLE 2:** Comparison of HVPG and EUS‐PPGM.

Parameter	HVPG	EUS‐PPGM
Measurement principle	Indirect: difference between wedged and free hepatic venous pressure	Direct: simultaneous portal vein and hepatic vein (or IVC) pressure measurement
Measurement target	Post‐sinusoidal vascular resistance	Portal vein pressure and porto‐systemic pressure gradient
Approach	Transvascular catheter technique (jugular or femoral access)	Transgastric/transhepatic EUS‐guided needle puncture
Invasiveness	Moderate (catheter‐based)	Low (endoscopic)
Radiation/contrast	Fluoroscopy and contrast required	Not required
Repeat measurement	Not easily feasible	Relatively feasible
Simultaneous procedures	Limited	EUS‐LB, EUS‐SWE, variceal assessment and treatment in single session
Utility in pre‐sinusoidal PH (e.g., MASLD and PSVD)	Limited: underestimates true portal pressure	Superior: direct measurement overcomes HVPG limitations
Current evidence level	Gold standard; extensive prospective data	Promising; accumulating data; multicenter trials needed

### 2.4 EUS‐guided Liver Biopsy

**FIGURE 4:**
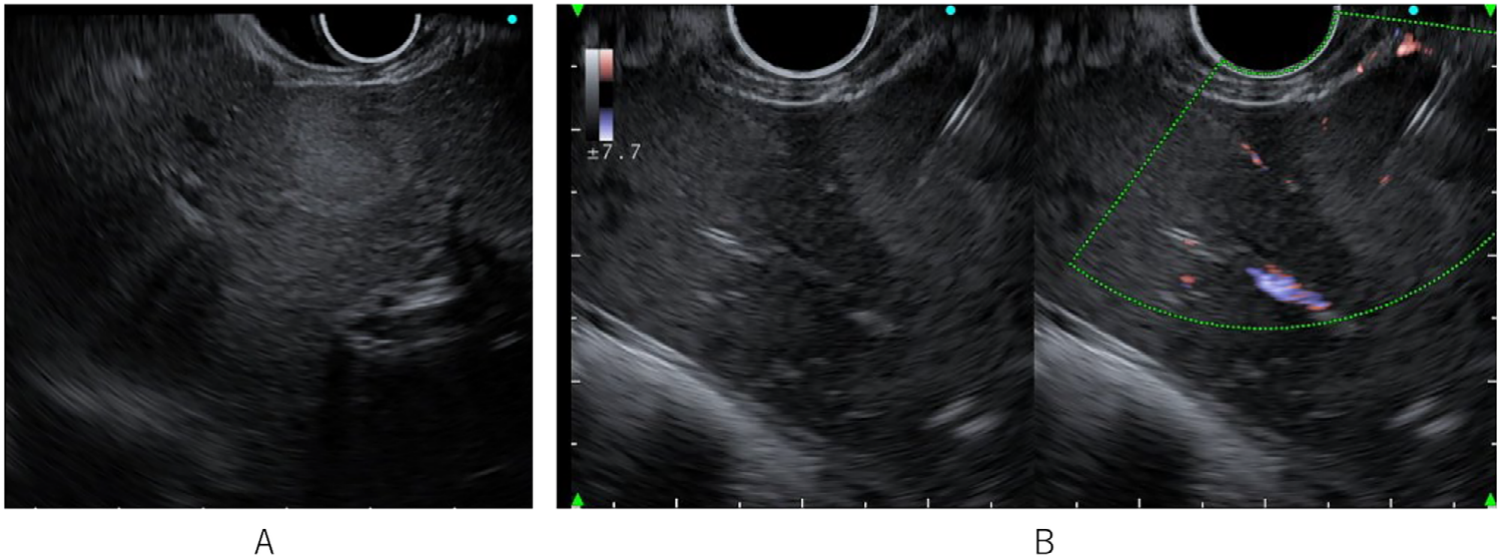
EUS‐guided liver biopsy. A: A hepatic tumor in the left lobe of the liver was visualized on an EUS image. B: Transgastric EUS‐guided tissue acquisition was performed. Liver biopsies for diffuse liver disease can also be performed using a similar technique.

EUS‐LB has emerged as a compelling alternative to percutaneous LB (PC‐LB), offering several distinct advantages: easy transgastric access to the left hepatic lobe under real‐time EUS guidance; minimal influence of obesity or ascites on specimen quality; the ability to sample both lobes in a single session for assessment of sampling variability; and the unique possibility of combining biopsy with EUS‐PPGM, EUS‐SWE, and variceal assessment within a single procedure [38] (Figure 4). These attributes position EUS‐LB as a natural cornerstone of the ‘one‐stop’ endo‐hepatology approach.

A 2019 meta‐analysis by Mohan et al. established overall diagnostic adequacy of 93.9% and an adverse event rate of 2.3% for EUS‐LB across 19 studies, confirming its safety and effectiveness [39]. Choi et al. found that EUS‐LB yielded significantly longer specimens than PC‐LB (*p* < 0.0001) with equivalent fibrosis staging accuracy [40]. A 2023 meta‐analysis by Chandan et al. including 748 patients showed marginally higher overall diagnostic accuracy for PC‐LB over EUS‐LB (98.6% vs. 88.3%, odds ratio: 1.65, *p* = 0.04). However, analysis restricted to RCTs revealed no significant difference, suggesting that needle‐type heterogeneity across studies largely explained the apparent gap [41]. A 2025 meta‐analysis of RCTs reported by Espirito Santo et al. (four trials, 258 patients) confirmed equivalent diagnostic adequacy, specimen length, portal tract counts, and adverse event rates between EUS‐LB and PC‐LB, with EUS‐LB associated with significantly lower post‐procedure pain (standardized mean difference −0.58, 95% confidence interval −0.95 to −0.22) [42].

A 2025 retrospective, cross‐sectional study by Xu et al. was the first reported study to include a three‐way morphologic comparison of EUS‐fine needle LB (EUS‐FNLB), PC‐LB, and transjugular LB (TJ‐LB) using American Association for the Study of Liver Diseases (AASLD) adequacy criteria (specimen length ≥2 cm and ≥11 complete portal tracts [CPTs]) and enrolled 160 patients: EUS‐FNLB 85, PC‐LB 50, and TJ‐LB 25 [43]. EUS‐FNLB demonstrated the greatest median aggregate specimen length (3.5 cm) and the highest median number of CPTs (18 CPTs) compared to either PC‐LB or TJ‐LB, with the highest rate of meeting AASLD adequacy thresholds. Specimen fragmentation was also evaluated as an additional morphologic parameter; EUS‐FNLB specimens showed favorable fragmentation characteristics. This study is particularly noteworthy because it extends the comparison beyond the conventional EUS‐LB versus PC‐LB paradigm to include TJ‐LB, which is the preferred approach for patients with coagulopathy or ascites. Moreover, this study demonstrates that EUS‐FNLB achieves superior morphologic specimen quality across all three comparators. Use of fine‐needle biopsy (FNB) needles over FNA needles for EUS‐LB is supported by a randomized trial showing superior specimen length and portal tract counts [44]. Taken together, findings of this study suggest that EUS‐LB is a safe and effective alternative to both PC‐LB and TJ‐LB: EUS‐LB offers superior tissue yield, lower post‐procedure pain, and the unique benefit of integration into a comprehensive single‐session endo‐hepatology assessment.

## 3 Therapeutic Endo‐Hepatology in PH

Reports of EUS‐guided vascular interventions (EUS‐VI) have increased considerably in recent years, reflecting both the maturation of interventional EUS techniques and growing recognition of their clinical value [45]. EUS‐VI relevant to PH management includes treatment of isolated gastric varices (GV), refractory EV, refractory hepatic encephalopathy via portosystemic shunt obliteration, and experimental procedures including intrahepatic portosystemic shunt (IPS) creation and portal vein stenting. Real‐time visualization of target vascular lesions is provided by EUS guidance, enabling precise needle and device placement that are beyond the capabilities of conventional endoscopic guidance alone, including identification of feeding vessels, measurement of variceal caliber, and confirmation of thrombosis by color Doppler [46]. Furthermore, EUS‐guided radiofrequency ablation (EUS‐RFA) for hepatocellular carcinoma (HCC) has emerged as an oncological application within the endo‐hepatology framework.

### 3.1 EUS‐guided Vascular Embolization

#### 3.1.1 Treatment of Gastrointestinal Varices

Although EIS and EVL remain the mainstays of EV treatment, some patients have complex hemodynamics, particularly patients with multiple large Pv feeding the varices. For such veins, which render conventional therapy technically challenging or prone to early recurrence, EUS guidance allows direct visualization and targeting of these hemodynamically important perforating vessels. Lahoti et al. first reported EUS‐guided EIS targeting esophageal Pv in 2000 [47]. More recently, Irisawa et al. reported successful EUS‐guided coil deployment combined with sclerotherapy for giant EVs that were difficult to control using conventional approaches, illustrating that EUS‐VI can serve as a rescue strategy in refractory cases [48].

For isolated GV, Romero‐Castro et al. first described EUS‐guided cyanoacrylate (CA) injection into perforating feeding veins in 2007 [49]. Given the risk of systemic CA embolization via spontaneous portosystemic shunts, including pulmonary embolism that is inherent in conventional endoscopic CA injection [50], the same group developed EUS‐guided coil embolization in 2010 [51]. Metallic coils sized to 120%–150% of the measured GV diameter deploy as a mechanical scaffold that markedly reduces systemic embolization risk. Binmoeller et al. subsequently combined coil placement with CA injection, demonstrating that the pre‐placed coil retains the CA within the varix and augments flow control while reducing the required glue quantity [46]. Long‐term outcomes in 152 patients over six years included technical success >99%, complete obliteration in 93%, and a rebleeding rate of only 3% in obliterated cases with no severe adverse event. Irisawa et al. reported a variant combining coil deployment with ethanolamine oleate (EO) injection, achieving durable obliteration in cases where EO reached the afferent vessels with no recurrence during follow‐up [52].

**FIGURE 5:**
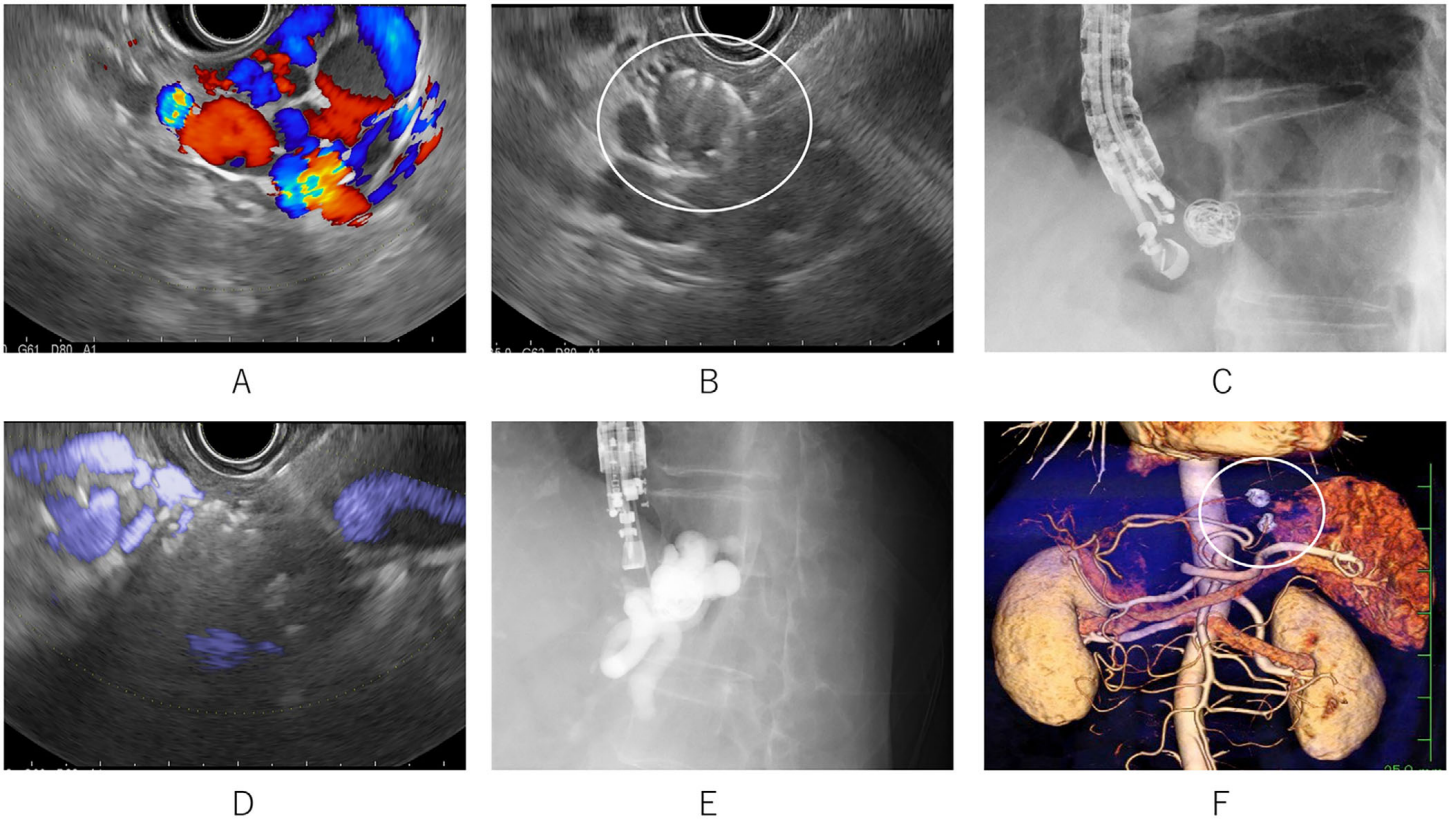
EUS‐guided coil deployment with sclerotherapy. A: Color Doppler EUS showed complex blood flow in the gastric varices. B: Coils were deployed and placed in the gastric varices under EUS guidance (area circled). C. Fluoroscopic image of the coil placement. D. Color Doppler showed that variceal blood flow was almost completely absent at the site where the coil was placed. E. After coil placement, a sclerosant (ethanolamine oleate) is injected to induce thrombosis of the remaining variceal lumen and blood supply pathway. F: Coils were placed at two sites, and the gastric varices completely disappeared (area circled).

A 2020 meta‐analysis by McCarty et al. confirmed EUS‐guided coil + CA combination therapy superiority over CA alone (technical success 98% vs. 96%, *p* < 0.001; adverse events 10% vs. 21%, *p* < 0.001) [53]. A 2025 meta‐analysis by de Mesquita et al. further demonstrated significantly lower recurrent bleeding and reintervention rates with EUS‐guided coil + CA versus endoscopic CA injection alone, with no difference in pulmonary embolism risk, definitively establishing the EUS combination approach as the preferred strategy [54]. EUS‐guided therapy has also been applied successfully to ectopic varices involving the duodenum [55, 56, 57, 58, 59] and rectum [60, 61], thereby extending the reach of endo‐hepatology beyond the traditional gastroesophageal distribution. Baveno VII explicitly lists EUS‐guided coil placement for varices as an area requiring further research, reflecting its growing clinical and scientific importance [12] (Figure 5).

#### 3.1.2 Treatment of Refractory Hepatic Encephalopathy

Large spontaneous portosystemic shunts, most commonly splenorenal or gastrorenal shunts, are a recognized cause of medically refractory hepatic encephalopathy that is difficult to manage with standard pharmacological therapy including lactulose and rifaximin. Shunt occlusion using balloon‐occluded retrograde transvenous obliteration or coil embolization via IVR is effective but technically demanding and not feasible when shunt anatomy precludes transvascular access. In 2023, Rathi et al. reported EUS‐guided transgastric portosystemic shunt obliteration using coils and CA in seven patients with recurrent hepatic encephalopathy in whom IVR had failed or was anatomically not feasible [62]. Complete Doppler‐confirmed flow occlusion was achieved in six of seven patients (86%), with significant clinical improvement in encephalopathy grade and with no severe adverse event. This study establishes EUS‐VI as a viable, minimally invasive alternative to IVR for portosystemic shunt obliteration in selected patients who have exhausted conventional therapeutic options.

### 3.2 EUS‐RFA for HCC

Since its establishment as a technique for ablation of pancreatic tumors including neuroendocrine tumors and insulinomas, EUS‐RFA has been explored for HCC when percutaneous RFA is limited by anatomically challenging lesion location (e.g., the hepatic dome, caudate lobe, or perivascular regions) or when intervening bowel, large vessels, diaphragm, or patient factors such as poor transcutaneous acoustic window preclude the percutaneous approach [63, 64]. Initial case reports from independent groups demonstrated both the technical feasibility and short‐term oncological efficacy of EUS‐RFA for HCC [65, 66]. Harwani et al. recently reported the largest EUS‐RFA series to date in five patients with HCC ≤30 mm, achieving technical success in all procedures without major adverse events such as liver abscess or hemorrhage [67]. These preliminary results are encouraging, but before clinical adoption, large‐scale prospective studies must be conducted with long‐term oncological endpoints including local recurrence‐free and overall survival.

### 3.3 EUS‐IPS Creation

For PH‐related complications including refractory ascites, hepatic hydrothorax, and prevention of variceal rebleeding, TIPS is an established and effective treatment [68]. EUS‐IPS creation, transgastrically deploying a stent between the left hepatic vein or inferior vena cava and the intrahepatic left portal branch, has been investigated in animal models as a potential minimally invasive complement to TIPS. In 2009, Buscaglia et al. first demonstrated the technical feasibility of EUS‐IPS in two pigs [69]. Poincloux et al. subsequently achieved successful stent placement in 19 of 21 pigs with confirmed PPG reduction [70]. No report of a clinical human study of EUS‐IPS exists. However, Zhang et al. described EUS‐guided coil marking of the portal vein to facilitate subsequent TIPS placement in a patient with cavernous portal vein transformation, a creative bridging application of EUS‐portal vein access that illustrates the technique's potential in anatomically complex cases [71].

### 3.4 EUS‐Guided Portal Vein Stenting

Portal vein stenosis causing symptomatic PH has been treated with percutaneous transjugular portal vein stenting in selected cases [72, 73]. Because EUS enables direct visualization and puncture of the extrahepatic portal vein, splenic vein, and superior mesenteric vein through the gastric wall, direct stent delivery without transjugular access is theoretically possible. Park et al. demonstrated successful uncovered metal stent deployment in the main portal vein of six pigs via a transgastric–transhepatic EUS‐guided approach without any significant complication or mortality, providing proof‐of‐concept for future clinical application for patients who are not suitable for conventional transjugular intervention [74].

## 4 Future Perspectives: “One‐Stop” Endo‐Hepatology

One of the most transformative concepts in endo‐hepatology is the ‘one‐stop’ comprehensive hepatological assessment within a single EUS session. Hajifathalian et al. demonstrated that EUS‐PPGM and EUS‐LB can be performed simultaneously and safely. Moreover, its PPG values and histological findings each independently correlate with clinically meaningful outcomes such as variceal presence and degree of hepatic decompensation, information which previously required separate hemodynamic catheterization and LB procedures [75]. Kim et al. extended this concept, demonstrating that EUS‐PPGM, EUS‐LB, and upper gastrointestinal variceal screening and treatment can all be completed in a single outpatient EUS session [76]. This “triple‐function” endo‐hepatology approach might fundamentally transform the initial hepatological workup from a multi‐visit, multi‐specialist process into a streamlined, patient‐friendly procedure with particular benefit in settings with limited specialist availability or barriers to follow‐up.

In their comprehensive review in 2025, Bruni et al. highlighted that the “one‐stop” approach provides diagnostic precision across a spectrum of PH etiologies, not only cirrhotic PH but also non‐cirrhotic forms such as PSVD where EUS‐PPGM's direct pressure measurement overcomes the fundamental limitations of HVPG [77]. Integration of EUS‐SWE into such a session could further add non‐invasive fibrosis staging without additional tissue sampling, making the comprehensive hepatological workup available in a single endoscopic procedure.

Several challenges must be addressed before endo‐hepatology procedures become universally accepted clinical standards. Standardization of techniques including needle selection, pressure measurement devices, durations of recording, and definitions of technical and clinical success remain as priorities across centers. Clear patient selection criteria and the identification of patients who benefit most from EUS‐PPGM versus HVPG will require evidence from prospective comparative studies. The thrombosis risk associated with direct portal vein puncture has been raised in the literature. Although existing clinical data have not revealed a high incidence, careful systematic prospective monitoring is warranted [78]. From a technological perspective, further development of miniaturized dedicated pressure transducers, wireless transmission systems, and automated waveform analysis software will be necessary for procedural standardization and widespread clinical adoption.

## 5 Conclusion

Endo‐hepatology represents a rapidly evolving interdisciplinary field bridging advanced endoscopy and hepatology, offering transformative diagnostic and therapeutic modalities for PH and CLD. EUS‐PPGM provides minimally invasive, direct portal pressure assessment with strong correlation to the gold‐standard HVPG, showing a unique clinically important advantage in pre‐sinusoidal PH disorders where HVPG is unreliable. EUS‐guided coil plus CA injection offers superior long‐term outcomes for GV compared to conventional endoscopic therapy, with a strong safety profile confirmed across multiple meta‐analyses. EUS‐LB provides tissue sampling equivalent in quality to PC‐LB with significantly lower post‐procedure pain and the unique advantage of integration into a comprehensive single‐session assessment. The “one‐stop” endo‐hepatology approach integrating EUS‐PPGM, EUS‐LB, liver stiffness assessment of fibrosis staging, and variceal evaluation and treatment in a single EUS session represents a paradigm shift with the potential to fundamentally transform the management of patients with CLD, reducing procedural burden and enabling more timely and comprehensive clinical decision‐making.

Mutual understanding, institutional collaboration, and shared decision‐making between endoscopists and hepatologists are essential prerequisites for the continued development and responsible dissemination of this field. Prospective multicenter trials, standardization of technique and reporting, and careful long‐term safety monitoring are critical to translate the promise of endo‐hepatology into universal clinical practice. We anticipate that endo‐hepatology, transcending traditional specialty boundaries, will contribute to next‐generation medical innovation and meaningfully improve outcomes for patients with PH and CLD, providing personalized management, avoiding progression of liver diseases, and offering a wide range of safe, accurate, and widely available procedures in daily clinical practice.

## Author Contributions

**Akira Yamamiya** and **Atsushi Irisawa** designed this manuscript, collected and analyzed the data, and drafted the manuscript; **Atsushi Irisawa** and **Rafael Romero‐Castro** checked the manuscript and approved the final version; **Yasunori Inaba** collected the literature and created the tables.

## Conflicts of Interest

Rafael Romero‐Castro is a consultant for Cook Medical. The other authors declare no conflicts of interest.

## Funding

The authors have nothing to report.
